# Bacteriostatic cells instead of bacteriostatic antibiotics?

**DOI:** 10.1128/mbio.02680-23

**Published:** 2023-12-21

**Authors:** Fernando Baquero, Jerónimo Rodríguez-Beltrán, Bruce R. Levin

**Affiliations:** 1 Department of Microbiology, Ramón y Cajal University Hospital, Ramón y Cajal Institute for Health Research (IRYCIS), Madrid, Spain; 2 Public Health Networking Biomedical Research Centre in Epidemiology and Public Health (CIBERESP), Madrid, Spain; 3 Public Health Networking Biomedical Research Centre in Infectious Diseases (CIBERINFEC), Madrid, Spain; 4 Department of Biology, Emory University, Atlanta, Georgia, USA; The University of Edinburgh, Edinburgh, United Kingdom

**Keywords:** bacteriostatic antibiotics, bacteriostatic cells, antibiotic mode of action

## Abstract

This year we commemorate the centennial of the birth of the mature concept of bacteriostasis by John W. Churchman at Cornell University Medical School. The term bacteriostasis has primarily been applied to antibiotics (bacteriostatic antibiotics). In this Opinion paper, we are revisiting this concept by suggesting that bacteriostasis essentially reflects a distinct cellular status (or "cell variant") characterized by the inability to be killed as a consequence of an antibiotic-induced stress impacting on bacterial physiology/metabolism (growth). Note that the term “bacteriostasis” should not be associated only with antimicrobials but with many stressful conditions. In that respect, the drug promotion of bacteriostasis might resemble other types of stress-induced cellular differentiation, such as sporulation, in which spores can be considered “bacteriostatic cells” or perhaps as persister bacteria, which can become “normal cells” again when the stressful conditions have abated.

**IMPORTANCE:**

This year we commemorate the centennial of the birth of the mature concept of bacteriostasis by John W. Churchman at Cornell University Medical School. The term bacteriostasis has primarily been applied to antibiotics (bacteriostatic antibiotics). In this Opinion paper, we are revisiting this concept by suggesting that some antibiotics are drugs that induce bacteria to become bacteriostatic. Cells that are unable to multiply, thereby preventing the antibiotic from exerting major lethal effects on them, are a variant (“different”) type of cells, bacteriostatic cells. Note that the term “bacteriostasis” should not be associated only with antimicrobials but with many stressful conditions. In that respect, the drug promotion of bacteriostasis might resemble other types of stress-induced cellular differentiation, such as sporulation, in which spores can be considered “bacteriostatic cells” or perhaps as persister bacteria, which can become “normal cells” again when the stressful conditions have abated.

## OBSERVATION

Bacteriostasis is generally understood as a property of a certain group of antibiotics’ action mode. In this Opinion paper, we here postulate that bacteriostasis might result from the bacterial cells’ changes that are promoted rather than generated by the antibiotic. Bacteriostatic antibiotics produce cells that cannot be killed. Since the first usage of the term more than a century ago, the notion of bacteriostasis has always been attributed to the physical challenges (such as bacteriostatic radiation) and the chemical compounds (bacteriostatic antimicrobial agents) exerting an inhibitory non-lethal effect on the growth of the tested microorganism. A century later, we can consider the possibility that bacteriostasis could result from bacteriostatic cells instead of bacteriostatic antibiotics. Bacteriostatic cells might be generated by various mechanisms, which are probably intertwined.

First, antibiotics might alter the bacterial metabolism and the construction and assembly of macromolecules, influencing the cells’ physical structure and surface/volume ratio, and thus its molecular interactions. Consequently, under antimicrobial exposure, the surviving cell is “a variant type of cell,” an altered cell that might be less susceptible to the lethal effect of antibiotic action. Second, the impact of antibiotic action on its target could trigger a metabolic challenge that results in halting cellular replication. Such a survival strategy is expected to occur as a response to other types of cellular stress, from nutritional stress to antimicrobial peptides and even to bacteriophages (1). Thus, “bacteriostatic antibiotics” are those that promote cellular changes leading to cell survival, whereas “bactericidal antibiotics” promote cellular changes resulting directly or indirectly in cell death (2).

### Bacteriostasis

The notion of bacteriostasis preceded the discovery of antibiotics and was applied a century ago, in 1923, to the observed antibacterial effect of basic dyes such as aniline or fuchsin; in the words of Churchman, “Organisms exposed to aniline dyes and seemingly killed may subsequently revive” (3). Following early 20th century studies by Marie Curie and Claudius Regaud, the term “bacteriostatic” was also used in the sense of “delayed death,” referring to the antimicrobial process resulting from radiation exposure to living bacteria and other types of cells (4). The concept of a time-dependent (delayed) process of bacterial inhibition or death resulting from antimicrobials’ primary mechanisms of action has recently been revisited (2).

**Fig 1:**
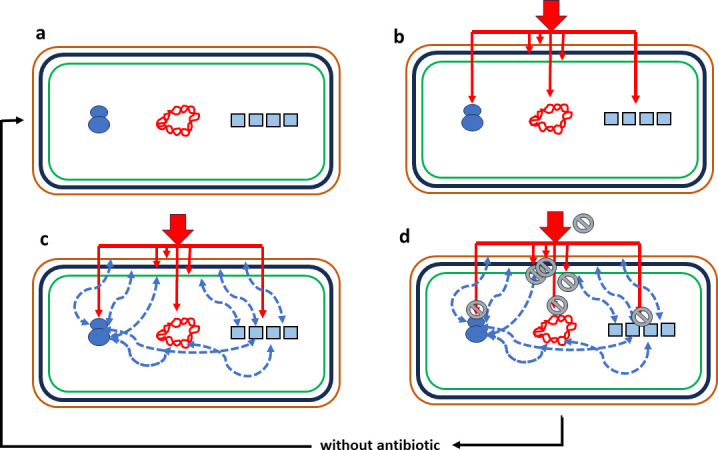
Antibiotics produce cellular bacteriostasis. (a) Schema of a bacterial cell with antibiotic targets: outer membrane (brown), cell wall (black), cytoplasmic membrane (green), ribosomes (blue), nucleoid (red, twisted), and metabolism (sequential blue squares). (b) The antibiotic exposure (thick red arrow) influences the function of their target(s) (thin red arrows). (c) The modification of target functioning influences other functions in the cell’s physiological interactive network (blue broken lines), producing a functionally altered cell. (d) Gray prohibitory signs represent the antibiotic’s inability to produce a lethal effect; the cells have been converted into bacteriostatic cells. If the antibiotic exposure is discontinued, the cell returns to its susceptible status (a).

Our hypothesis is that bacteriostasis is related to the inability to be killed due to the modified status of cellular physiology and metabolism after antibiotic exposure (or other sources of stress) more than to the primary antibiotic mode of action. Cells under slow growth or in a stationary phase are frequently refractory to being killed by most bactericidal agents; they adopt a “bacteriostatic mode.” An exception are those antibiotics (or antimicrobial peptides) that directly damage the integrity of the cells’ physical structure, such as bacterial membranes, or that promote the denaturation of nucleic acids (2). The move to a cellular “bacteriostatic mode” is a consequence of confronting various types of stress, including nutritional stress. This situation is common in natural bacterial life (such as *Escherichia coli* outside the culture tube): the “famine” waiting for the “feast” (5). When cellular division is impaired, bacteriostasis (which is difficult to distinguish from very slow growth) happens. This occurs in diversely generated slow or nonreplicating bacteria, due to a shortage of nutrients and resources; in slow-growing cells derived from metabolic changes, as persisters, sometimes considered to be involved in “drug-induced bacteriostasis” (6); or in antibiotic-static cells when antibiotics interfere with essential cellular processes (7). The concept of “antibiotic-static cells” proposed in the above study might resemble the concept of “bacteriostatic cells” driven by antibiotic exposure, suggesting the induction of a process of cellular reorganization, thus involving structural changes in shape and functional changes in metabolism (7). These “new types of cells” are more resistant to many antibiotic agents and phages, starvation, and to physical and chemical aggressions. For example, bacteriostatic antibiotics such as azithromycin or linezolid promote the emergence of “antibiotic-static cells” that do not support the replication of most phages in *Staphylococcus aureus* (8). In addition, the time gained by reducing cellular replication speed after bacteriostatic antibiotic treatment allows cells to acquire immunity to phages via CRISPR-Cas systems, suggesting that bacteriostasis might also be adaptive beyond antibiotic resistance (9). A schema of our hypothesis of “cellular bacteriostasis” is presented in Fig. 1. Finally, cellular bacteriostasis might have clinical consequences, as non-growing bacteria might reduce the immune response. The local release of “pathogen-associated molecule patterns” (PAMS), which are recognized in pattern-recognition receptors of macrophages and dendritic cells, driving an effective immune response, is reduced under bacteriostasis. Such an effect can be reinforced in biofilm slow-growing cells, where the production of extracellular polymeric substances reduces PAMS exposure and thus the immune response. Immunocompromised hosts, where the bacteriostatic survivors are spared from killing, should be more affected by the emergence of bacteriostatic cells than the immunocompetent ones.

### Sporulation as a process of bacterial cell differentiation leading to cells with a resistant bacteriostatic phenotype

The formation of these altered cells resembles the biological phenomenon of cellular differentiation, in which two cells with identical genomes differ in their growth, physiological, and resilience functions, and such differentiation is reversible. Sporulation is the classic example of cellular differentiation in stressed bacteria, which leads to enhanced survival conditions. Sporulation consists of the production, as a response to stress conditions, of a “new type of cell” with an identical genome to the mother vegetative form, which is transiently preserved from being killed, thereby constituting a “bacteriostatic cell.” How frequent is sporulation in bacteria? The *spo* genes, whose transcription is triggered by a drop in intracellular GTP and govern the formation of endospores, are only found in the two major classes of Firmicutes (Bacillota): Bacilli and Clostridia, and in closely related organisms (10). Bacillota spores result from the asymmetric division of a cell, in which one of the daughter cells forms a different type of cell: the endospore (11), an almost completely dormant cell (nearly zero metabolism). Similar stress-induced cellular differentiation occurs in *Myxococcus*, a soil bacterium producing multicellular fruiting bodies filled with environmentally resistant spores. The spores (myxospores) differ from Bacillota endospores in that they are formed by the morphogenesis of whole cells; cells within fruiting bodies become spores (12), and these myxospores retain some metabolic activity. In the sporulating Actinobacteria, filamentous bacterial cells (as in the case of *Streptomyces*) form chains of spores, also keeping some metabolic activity and even with functional flagella. Even in Archaea, there is cellular differentiation into hyphae and spores (13). This consideration indicates that the formation of cellular differentiation, that is, the emergence of “different types of cells,” is a widespread strategy in the microbial world to ensure the resilience of bacterial populations when confronting different types of stress, while also ensuring a reversion to more reproductive forms after the disappearance of life-threatening conditions.

### Are there other types of cellular differentiation that promote survival under stress conditions?

A similar process of cellular differentiation might occur in other bacterial organisms, including non-sporulating Firmicutes, such as *Staphylococcus* or *Enterococcus*, or in Gram-negative ones, such as Gammaproteobacteria, including *E. coli*, *Acinetobacter*, and *Pseudomonas*. Small colony variants (SCVs) in *Staphylococcus* could represent an alternative escape strategy resulting in an analogous response that renders the cells bacteriostatic to the endospore production of spore-forming bacilli. In fact, the biochemical (not necessarily the genetic) mechanisms resulting in SCV formation resemble those involved in the sporulation process. In both *Staphylococcus* and *Bacillus*, non-sporulating and sporulating organisms, respectively, stress-induced “cellular differentiation” involves changes in menadione (a lipid-soluble vitamin influencing the cell membrane structure) biosynthesis, the reduction of which promotes SCV formation or sporulation, respectively. That process indicates that similar biochemical strategies are used for both processes of differentiation. Antibiotic-associated persistence could also be considered a form of cellular differentiation in response to potentially lethal stressors that generate errors in DNA replication (14). Also, the toxin-antitoxin systems might contribute to bacterial protection from killing (15). They might play a role in the formation of “persisters,” together with the SOS response (6). In *Bacillus*, conditions initiating sporulation (typically nutritional starvation) produce a partial deprivation of intracellular amino acids, fostering a stringent response. Amino-acid-uncharged tRNA stimulates the ribosome-associated protein RelA to synthesize the hyperphosphorylated guanosine nucleotides (p)ppGpp, relocating bacterial resources, with a resulting reduction in growth rate and in biosynthetic processes. In summary, several mechanisms are convergent to ensure that less metabolically active cells are more resistant, regardless of the potentially lethal perturbation.

### The bacteriostatic cell variants

Bacteriostatic cell variants (BCVs) are cells with a distinct morphology, resulting from an altered cell metabolism, which arise without genetic changes from their original populations when challenged by environmental stresses, including antibiotic exposure. They exhibit increased resistance to these potentially lethal conditions, and they have the capability of reversion to normal cells when these stress conditions are no longer present. This definition fits with all types of spores. In the case of non-sporulating organisms, something similar occurs during conventional sporulation, including cellular morphological changes. In *Staphylococcus* and *Enterococcus*, the SCVs, which we consider a type of BCV, have gross morphological alterations, with larger cocci. These cells frequently divide asymmetrically, similar to what generally happens in sporulating bacteria (16). In *E. coli*, SCV cells are swollen and elongated, and as in the case of Firmicutes, they frequently show cellular constrictions typical of incomplete or asymmetrical cell division (17).

### Antibiotic exposure and bacterial differentiation

Spore-forming *Streptomyces*, *Bacillus*, *and Myxococcus* frequently produce natural antibiotics, and this production is usually concomitant with cellular differentiation. Actually, in the case of the *Streptomyces*, antibiotic production occurs in the substrate mycelium, prior to aerial mycelium formation and sporulation. A similar process might occur in *Bacillus*, producing antibiotics that facilitate biofilm formation at sub-inhibitory concentrations. In such structured environments, the synthesis of other antibiotics could be involved in the action against non-sporulating sister cells (cannibalism). This can also be the case for *Myxococcus*, where sporulation timing follows a previous process of aggregation, which is followed by antibiotic production and spore formation, where a number of vegetative cells directly transform into spores.

This probably ensures that the nutritional resources released by the cell lysis of sporulating organisms are invested efficiently in the expensive process of spore construction and are not consumed by local competitors of other species. All spore-forming organisms produce antibiotics following stress, most frequently nutritional and oxidative stress (18). These are the same conditions pushing the bacterial population to sporulate. Both processes are intertwined, but non-antibiotic-producing mutants can sporulate (19). How do antibiotics contribute to the sporulation process? Under certain conditions, antibiotics, particularly those impairing protein or RNA synthesis, could restrict sporulation initiation, probably because they interfere in the production of stringent response mediators, such as RelA, and consequently (p)ppGpp. On the other hand, antibiotics could contribute to the metabolic rewiring leading to bacteriostasis. From a population perspective, the process of producing altered variant cells in a state of stasis (dormancy) is energetically costly. Antibiotics might reduce the number of reproducing cells in the population to lower the consumption of the remaining nutrients and ensure the feeding of the spore-forming survivors. As suggested by the above examples about *Streptomyces* and *Bacillus*, antibiotic (and probably biofilm niche construction) produced by the cell’s majority facilitates a high local nutrient concentration, which facilitates the emergence of the minority population of cells that will go on to sporulate (20). Probably that might promote genetic bottlenecks, which could explain that sporulation rate might evolve in specific populations submitted to frequent stress conditions.

### Antibiotic exposure and the genesis of bacteriostatic cell variants

As explained in previous sections, antibiotics influence the emergence of differentiated types of stress-tolerant cells that can persist during harsh conditions. There is a difference between the function of antibiotics in natural settings and the consequences of exposure to antibiotic drugs. In nature, antibiotics might play a role as a signaling agent (21)*,* and we cannot reject the hypothesis that this signal might foster the emergence of stress-resistant populations based on BCVs. Bacterial cells have a limited ability to identify the specific type of stress that they are experiencing, which explains why the bacteriostatic cells’ emerging variants have a broad spectrum of resistance. On the other hand, antimicrobial agents distort bacterial metabolism and the construction of macromolecules, so that under antimicrobial exposure, the surviving cell becomes a different, altered type of cell. As a known example, bacteria exposed to beta-lactams result in altered cell shapes, depending on the depending on the penicillin-binding-proteins (PBPs) that are inhibited. Spherical *E. coli* cells are promoted by mecillinam, and filamented cells by aztreonam; in both cases, these cells are more resistant to being killed (22).

Any change in the cell structure, shape, or biosynthetic processes could have functional alterations resulting from changes in the internal (physical) connectivity of membranes, ribosomes, proteins, RNAs, and the nucleoid. The shape can affect the cellular surface/volume ratio, with consequences in the local concentrations of biomolecules and their functional interactions. This phenomenon has been named “structural epistasis” (23). New organizations in the bacterial subcellular network could produce bacteriostatic cell variants. Antibiotics increase the proportion of SCVs in bacterial cultures. In *E. coli*, exposure to so-called bacteriostatic agents (such as chloramphenicol or azithromycin) gives rise to the emergence of SCVs (data not shown). In *S. aureus*, thymidine-dependent SCVs acquire morphological changes like those induced by trimethoprim exposure (24) and, as in the case of sporulation, there is a spontaneous increase in SCV production during the prolonged stationary phase. So-called bacteriostatic antibiotics produce a stationary phase, pushing cells toward a bacteriostatic mode in which they cannot exert any significant loss in viability, probably increasing longevity and assuring growth (25).

In agreement with the main hypothesis, one would expect different bacterial species to show different responses (i.e., bacteriostatic or bactericidal) to the same antibiotic. This is the case with some macrolides (e.g., erythromycin, azithromycin, and clarithromycin), classic bacteriostatic antibiotics that are indeed bactericidal against *Streptococcus pyogenes* and *Streptococcus pneumoniae* but not against *Staphylococcus aureus.* Similarly, *c*hloramphenicol is bactericidal at clinically relevant concentrations against *Haemophilus influenzae, Streptococcus pneumoniae*, and *Neisseria meningitidis* but produces a bacteriostatic effect in the bacteria of the Enterobacteriaceae family. Finally, staphylococci and enterococci show a bacteriostatic response when treated with linezolid, while streptococci are killed rapidly (26). These heterogeneous responses to antibiotic treatment are a manifestation of the various metabolic and physiological strategies deployed across the bacterial phylogeny and support the idea this Opinion paper intends to illustrate: a possible shift from the notion of “bacteriostatic antibiotics” to that of “bacteriostatic cells” resulting from antibiotic exposure. In such a view, the cell is the bacteriostatic object, not the antibiotic. Such a conceptual change blurs the distinction between bacteriostatic and bactericidal antibiotics, which often share similar mechanisms of action. The importance of this new notion is that we should consider exploring and eventually enhancing the bactericidal effect of some drugs considered bacteriostatic by tuning the bacterial physiological network. In addition, we should evaluate the antibiotic susceptibility of the “bacteriostatic cells arising from antibiotic exposure,” given that this exploration might provide clues achieving new modes of non-mutation-based collateral sensitivity with potential clinical interest for the design of successive antibiotic treatments for infections.
